# Myotonic Dystrophies 1 and 2: Complex Diseases with Complex Mechanisms

**DOI:** 10.2174/138920210790886844

**Published:** 2010-04

**Authors:** Benedikt Schoser, Lubov Timchenko

**Affiliations:** 1 Department of Neurology, Friedrich-Baur Institute, Ludwig-Maximilians-University, Ziemssenstr. 1a, Munich, Germany; 2 Department of Molecular Physiology and Biophysics, Baylor College of Medicine, One Baylor Plaza, Houston, TX 77030, USA

## Abstract

Two multi-system disorders, Myotonic Dystrophies type 1 and type 2 (DM1 and DM2), are complex neuromuscular diseases caused by an accumulation of expanded, non-coding RNAs, containing repetitive CUG and CCUG elements. Similarities of these mutations suggest similar mechanisms for both diseases. The expanded CUGn and CCUGn RNAs mainly target two RNA binding proteins, MBNL1 and CUGBP1, elevating levels of CUGBP1 and reducing levels of MBNL1. These alterations change processing of RNAs that are regulated by these proteins. Whereas overall toxicity of CUGn/CCUGn RNAs on RNA homeostasis in DM cells has been proven, the mechanisms which make these RNAs toxic remain illusive. A current view is that the toxicity of RNA CUGn and CCUGn is associated exclusively with global mis-splicing in DM patients. However, a growing number of new findings show that the expansion of CUGn and CCUGn RNAs mis-regulates several additional pathways in nuclei and cytoplasm of cells from patients with DM1 and DM2. The purpose of this review is to discuss the similarities and differences in the clinical presentation and molecular genetics of both diseases. We will also discuss the complexity of the molecular abnormalities in DM1 and DM2 caused by CUG and CCUG repeats and will summarize the outcomes of the toxicity of CUG and CCUG repeats.

## INTRODUCTION

Myotonic dystrophies (DMs) are autosomal dominant, multisystem diseases with a common pattern of clinical signs and symptoms such as myotonia, muscular dystrophy, cardiac conduction defects, cataracts, and endocrine disorders. In 1909, the German Steinert and the British doctors Batten and Gibb described the “classic” type of myotonic dystrophy (DM1, OMIM 160900) [1,2]. Later in 1912, uncovering high frequencies of familial cataracts in DM1 led to the recognition of DM1 as “multisystem disorder” [3,4]. DM1 is characterized by genetic anticipation. This term was introduced in 1918 to define lowering of the age of onset and aggravation of the disease in consecutive generations [5]. In 1994, a different multisystem disorder was identified with comparable clinical features to DM1 but with missing the DM1 CTG repeat expansion [6,7]. In Europe, the disease has been termed “Proximal Myotonic Myopathy” (PROMM, OMIM 160900), and in the United States “myotonic dystrophy with no CTG repeat expansion”. After a genetic discovery of the PROMM mutation in 2001 [8], and re-classification [9], the myotonic dystrophies are now termed DM1 for the classic type, and DM2 for all other late-onset proximal myotonia with the DM2 mutation. Both types of DM belong to the group of most variable human disorders distressing almost all systems of the human body.

## CLINICAL PRESENTATION OF DM1 AND DM2

### DM1: Congenital form of Disease

Congenital Myotonic Dystrophy (CDM) exists only in DM1. In 1960, Dr. Vanier has described 6 children with DM1 (the youngest one was 9 months old) with the disease manifestation at the time of birth [10]. After this first milestone description, additional case reports and larger series gave a more comprehensive description of the CDM phenotypic variation [reviewed in 4]. CDM onset varies from the pre-/postnatal period to adulthood. Within a wide clinical spectrum of CDM, there are some separate phenotypes according to the age of onset and the number of copies of CTG repeats. Genotype phenotype correlations in CDM showed an insignificant raise of CTG repeats in the juvenile-adult form of disease with the longer expansions in patients with childhood onset [4]. The largest CTG repeat expansions (> 1,500) are observed in patients with CDM [4].

Because of the large expansions of CTG repeats, CDM children may be born as premature infants. In many reported pregnancies, fetal movements are reduced and polyhydramnios occurred. Postnatal hypotonia and immobility are important first symptoms of CDM. In up to 50% of CDM, bilateral talipes and other contractures are present at birth. Facial diplegia with a tent-formed upper lip and a high arched palate is a characteristic feature. This weakness causes a weak cry and the inability to suck in approximately 75% of affected newborns. In survivors hypotonia is steadily improving and only rarely prominent at 3–4 years of age, but facial diplegia leads to the typical facial “carp-mouth” appearance. Respiratory complications are frequent in neonates. Severely affected neonates requiring ventilation for more than 4 weeks will die from respiratory problems. Delayed motor development is an important feature at postnatal stages. Almost all children become able to walk independently. Mental retardation is observed to a variable degree in a great number of affected individuals but normal mental development is possible, even if motor development is delayed. Rarely, attention deficit hyperactivity and anxiety disorders, autism, behavioural problems, and depression are reported in childhood. Despite the severe muscular phenotype, clinical myotonia is neither a feature presented in the neonatal period nor can it be disclosed in the electromyogram (EMG). Furthermore there is a high frequency for other associated abnormalities such as inguinal or hiatus hernia, undescended testis, congenital dislocation of the hip and heart defect, hydrocephalus, congenital cataract, and cleft lip [4, 11].

### Phenotype of Adult DM1 

**Fig. (1):**
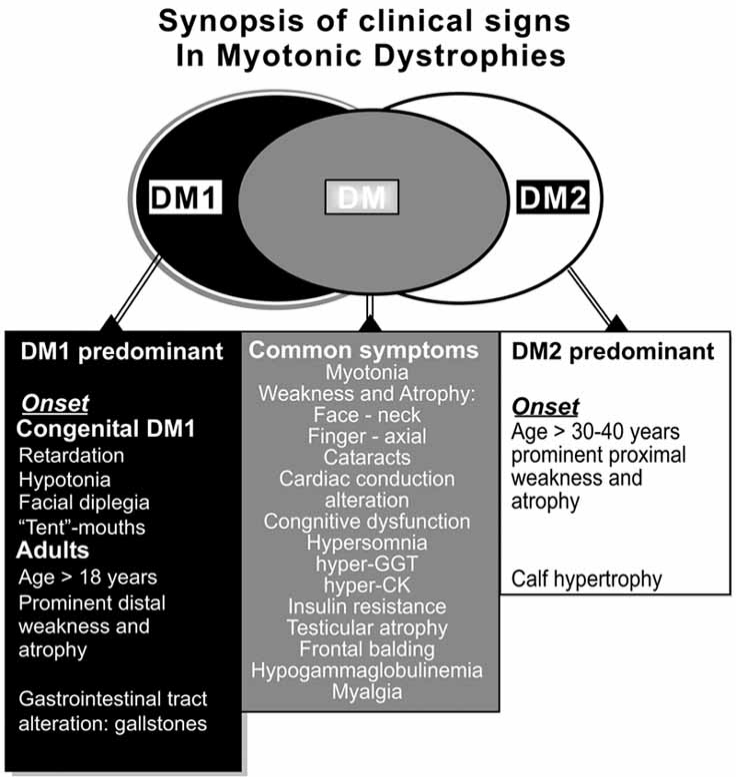
Synopsis of clinical signs in DM1 and in DM2.

**Fig. (2):**
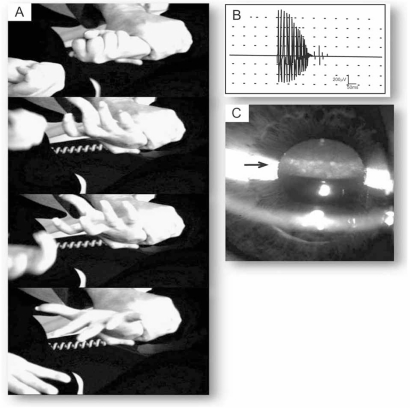
**Common clinical presentation in DM1 and in DM2.** (**A**) Grip myotonia in a DM2 patient. Reduced speed of first opening and weakness of the finger flexors. (**B**) Electromyographic recording of the tibialis anterior muscle from a DM1 patient with a classic myotonic discharge. Note the decrescendo character of the spontaneous motor unit activity. (**C**) Representative posterior iridescent cataract in a DM2 patient.

**Fig. (3):**
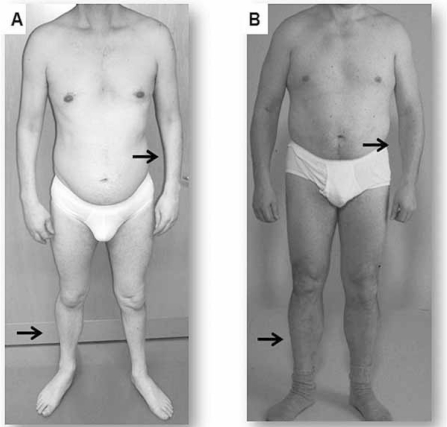
**Differences in clinical presentation of adult DM1 and DM2.** A classic forearm atrophy is shown for patient with DM1 (**A**) but not with DM2 (**B**). The “core” characteristic of DM2 is a typical predominant lower leg weakness and atrophy (**B**).

The adult onset clinical phenotype is the most typical appearance of DMs. The core features are facial weakness with ptosis and distal muscle weakness. Grip and percussion myotonia are regular features; however, myotonia affects any other muscle including bulbar, tongue or facial muscles, causing problems with talking, chewing, and swallowing. Furthermore, a 3- to 7-fold elevation of the serum creatine kinase is apparent. Cardiac involvement includes conduction abnormalities with arrhythmia and conduction blocks up to sudden cardiac death. In some patients and families, a dilated cardiomyopathy may be observed. Central nervous system involvement covers cognitive impairment/mental retardation, specific patterns of psychological dysfunction, personality traits, neuropsychological changes, and excessive daytime sleepiness. Some of these features may be related to alterations found by neuroimaging and neuropathology (e.g. tau protein abnormalities). The most common eye defect are the posterior capsular cataracts and, rarely, pigmentary retinal degeneration. Gastrointestinal tract involvement covers irritable bowel syndrome and symptomatic gall stones and, gamma-glutamyltransferase elevations. Finally, endocrine abnormalities include testicular atrophy, hypotestosteronism, insulin resistance with usually mild type-2 diabetes (Fig. **1-3**) [4, 11].

### DM1 Aging, Prognosis, and Outcome

By aging, more proximal and axial muscle weakness is common and many patients are wheelchair bound. Nevertheless, myotonia decreases by progressive muscle atrophy. Late, but prominent respiratory insufficiency occurs by diaphragmatic weakness. Still, cognitive impairment increases. The majority of DM1 patients have overt type-2 diabetes at older ages. Thus, in summary, many patients become severely disabled by their fifth and sixth decades of life. Chest infections, partly by aspiration, and diaphragm weakness are common and may precipitate respiratory failure. Sudden cardiac death is not uncommon, even in younger patients, and may be preventable, at least in part, by cardiac pacemaker implantation. On the other side, especially in late-onset or asymptomatic patients (with low number of CTG repeats), only limited features are found on clinical and paraclinical assessment. In late-onset patients, the search for cataracts is helpful for identifying the transmitting person [4,11].

### Clinical Phenotype of DM2

The most important discrepancy between DM1 and DM2 is absence of a congenital or early-onset form in DM2 [9, 11,12]. Exercise-induced fatigue and myalgia and/or mild grip myotonia and myotonia of the proximal legs, e.g. while climbing a staircase, and sometimes early neck flexor weakness are the most frequent early symptoms in patients affected with DM2. A core pattern of DM2 phenotype is proximal leg weakness and stiffness, deep finger and neck flexor weakness that appear between the fourth and sixth decades of patients’ lifespan. Patients have difficulties rising from squat, climbing stairs or rising from a chair. Muscle atrophy is only mild and almost never seen in the forearm. In some patients, muscle hypertrophy of the calves may be noticed. Highly patchy and temporary, abundant myalgia is regular. Mild to moderate, but even very high elevation of the serum creatine kinase may be found. Cardiac involvement with mild arrhythmia and conduction blocks up to sudden cardiac death can be noticed [11,13]. Progressive dilated cardiomyopathy seems to be more common in DM2 than in DM1. Central nervous system involvement covers mild cognitive dysfunctions, some psychological dysfunction, abnormal personality traits, neuropsychological changes and daytime sleepiness. Like in DM1, these findings may be associated with the reported alterations found by neuroimaging and may be connected to tau protein abnormalities [14]. Most common feature of DM2 phenotype is posterior capsular cataracts. Gastrointestinal tract involvement is limited, but elevations of gamma-glutamyltransferase levels are frequently noticed [11,15]. Endocrine abnormalities consist of insulin resistance with mild type-2 diabetes, hypothyroidism, hypotestosteronism, and rarely testicular hypotrophy (Fig. **1-3**).

### DM2 Aging, Prognosis and Outcome

By aging, more axial and distal muscle weakness is common and some patients are wheelchair bound. Myotonia decreases by progressive muscle atrophy. Even late, there is seems no overt respiratory insufficiency. Cognitive impairment increases very slowly. Only some of DM2 patients have overt diabetes at older age. Thus, in summary, only few patients become severely disabled by the sixth to eighth decades. However, there are seems to be many asymptomatic and undiscovered DM2 patients. Even by careful clinical and paraclinical assessment, sometimes it is challenging to recognize DM2 phenotype [11,15]. Thus, future collection of clinical data will show how DM2 mutation affects aging.

### How to Make the Correct Clinical Diagnosis in both DM?

#### DM1

The obvious clinical phenotype and the family history helps the diagnosis. In late-onset patients, different specialists may be involved in the treatment of symptoms. Genetic analysis is used to identify and/or confirm the diagnosis. Therefore, muscle biopsy is only rarely required. However, muscle biopsies are required in cases with neuromuscular complaints and with negative genetic analysis [11,16].

#### DM2

Different specialists may initially be involved in diagnostics. When proximal weakness or myotonia becomes obvious, together with a positive family history, diagnosis can be made. Genetic analysis is advised to confirm the diagnosis. A muscle biopsy may be required in asymptomatic neuromuscular patients or when genetic analysis for DM1 and DM2 is negative.

## MOLECULAR GENETICS IN DM

In 1992, DM1 mutation has been discovered on chromosome 19q as an expansion of CTG repeats in the 3’ untranslated region of the dystrophia myotonica-protein kinase (*DMPK*) gene [4,15,17,18]. The incidence of DM1 is 13: 100,000, the prevalence 2-5 / 100,000, in the congenital form 1: 3,500 [4,11,15]. The length of CTG expansions varies from 80 to more than 4,000 repeats in affected patients, and the length of expansion is from 50 to 100 CTG repeats in asymptomatic patients. Somatic instability has been reported in different tissues over lifespan with repeat size elongation of ~50-80 repeats per year. Intergenerational instability is frequent, with expansions of several thousand repeats occurring in a single generation, most commonly secondary to maternal transmission [4,11,15]. There is a relative correlation between the length of CTG repeat expansions and age of onset for DM1 patients with CTGs <400, but correlation between repeat length and disease severity is poor for long repeats [4,11,15] (Table **1**).

In 1998, the DM2 locus was mapped to 3q21 and, thereafter, the mutation was identified as a CCTG expansion in intron 1 of the zinc finger protein 9 (*ZNF9*) gene [19, 20]. On the expanded alleles, an uninterrupted variant of the CCTG portion of the repeat tract is elongated [19] (Table **1**). Expanded DM2 alleles show extraordinary somatic instability with significant increases in length over time (e.g. 2000 bp/3 years) and expanded alleles often appear as smears by Southern blotting analysis [8,21]. The incidence and prevalence of DM2 seems to be in the range of DM1 (1:10,000). Haplotype analyses of DM2 families suggested that the DM2 expansions may have originated from one or more founder mutations and showed evidence that the normal repeat tract found in humans has a conserved biological function [21,22]. The age of the founding haplotype and the DM2 (CCTG) expansion mutation is estimated at approximately 200 to 540 generations [21]. A family of apparently Afghan ancestry provides some confirmation that the DM2 expansion occurred previous to Aryan migration of Indo-European settled Aryana (ancient Afghanistan) in 2000-1000 B.C [21,23].

## DM1 AND DM2 PATHOLOGIES ARE ASSOCIATED WITH TOXICITY OF CUG AND CCUG RNA REPEATS 

### The Processing and Fate of the Mutant DMPK mRNA in DM1 Cells

The experimental work during last 15 years has been focused on the molecular mechanisms by which expansions of CTG and CCTG repeats cause DM phenotype. Although CTG repeats affect expression of DMPK protein and the transcription of genes in the DMPK locus (recently reviewed in details in [24]), multisystemic nature of DM1 is mainly associated with the accumulation of non-coding CUG repeat RNA [25-34]. Thus, we will focus this review on the role of CUG and CCUG repeats in DM pathology. Numerous observations present strong evidence that large amounts of CUG and CCUG non-coding RNAs are toxic for cell functions. Several hypotheses have been suggested addressing mechanisms of the toxicity of expanded non-coding CUG and CCUG repeats.

Early studies of DM1 mechanisms have investigated the transcription and post-transcriptional processing of the mutant DMPK RNA in DM1 patients. The main hypothesis of these studies was that the expanded RNA CTG repeats in the 3’ UTR of DMPK may interfere with transcription, post-transcriptional modifications (splicing, adenylation and cap formation) and export of the mutant DMPK mRNA from nucleus to cytoplasm. It has been shown that the mutant DMPK mRNA changed processing of wild type DMPK mRNA through trans effect presenting the first evidence for the toxic role of CUG RNA repeats in DM1 pathology [25]. At the same time, the levels of DMPK mRNA varied from patient-to-patient [35]. No significant changes in the mutant DMPK mRNA stability and polyadenylation have been found [36]. Data for the nucleus/cytoplasmic localization of mutant DMPK mRNA remain controversial. The pioneering study from Dr. Singer’s laboratory showed that the mutant DMPK transcripts form large aggregates or foci in nuclei of DM1 fibroblasts and in DM1 tissue [26]. However, the formation of nuclear foci did not seem to interfere with the export of mutant DMPK mRNA from nuclei to cytoplasm [26]. Large amounts of the mutant DMPK mRNA have been detected in cytoplasm of DM1 fibroblasts. In contrast to the nuclear foci, the cytoplasmic mutant DMPK mRNA was detected in small complexes [26]. Other studies demonstrated that the mutant DMPK transcripts are completely blocked in nuclei [37,38]. In addition to the aggregated form of mutant DMPK, we have also detected non-aggregated forms of the mutant CUG RNA [39]. Other studies also suggested the existence of un-aggregated (soluble) form of the mutant DMPK mRNA in DM1 nuclei [40, 41].

Several studies have examined common mechanisms of aggregation of RNAs. The aggregation of RNAs in nuclei of *S. cerevisiae *has been shown upon block of RNA nuclear export [42]. Such retention of the nuclear RNAs in *S. cerevisiae* required the components of the nuclear exosome including protein RRP6 since deletion of the RRP6 gene releases trapped RNAs from intranuclear foci [43]. One might assume that, by analogy to these observations, the aggregation of the mutant DMPK mRNA in nuclei is likely mediated by a reduced processing of the mutant DMPK mRNA. Such reduction of the mutant DMPK mRNA export could be because of diminished capacity of the modification enzymes to process the mutant DMPK mRNA. In agreement with this hypothesis, it has been shown that a truncated DMPK RNA containing long CUG and CAG repeats aggregated in nuclear foci; likely due to delay in the processing of transcripts with expanded repeats [44]. Two separate studies from Dr. Lawrence’ lab and from Dr. Morris’ lab have examined the fate of mutant DMPK RNA in DM1 nuclei. They found that the mutant DMPK mRNA is located at the periphery of nuclear speckles, suggesting that the efficiency of export of the mutant DMPK pre-mRNA is reduced [45,46].

Despite the nuclear aggregation of the mutant DMPK mRNA, a significant portion of this mRNA is still transported to cytoplasm [26,39,47]. It has been recently found that, in DM1 cells, the mutant DMPK mRNA is detected in aggregated forms in both nucleus and in cytoplasm [47]. Approximately 23 to 71% of DM1 fibroblasts and myoblasts contain CUG foci in both nuclei and in cytoplasm [47]. Identification of the mutant DMPK in aggregated form in cytoplasm of DM1 cells suggests that, like in nuclei, cytoplasmic processing of mutant DMPK mRNA might be also reduced by the CUG repeat tract. Although cytoplasmic foci do not affect splicing activity in DM1 cells, the role of cytoplasmic CUG foci in the regulation of translation or stability of RNAs has not been studied.

One of the possible reasons for the existing controversies on the effect of CUG repeats on mutant DMPK nuclear-cytoplasmic export might be the sensitivity of assays. The initial studies showed that the sensitivity of FISH assay is very important because cytoplasmic mutant DMPK mRNA is detected in the multiple complexes of smaller size relatively to very large nuclear foci [26]. It is important to note that recent progress in the detection of microRNAs showed that application of locked nucleic acid (LNA), modified oligonucleotides for hybridization with microRNAs, significantly increases sensititivity and specificity of hybridization [48]. Indeed, application of LNA-CAG probe and inclusion of Triton X-100 in the hybridization buffer for detection of mutant DMPK mRNA revealed multiple cytoplasmic aggregates of mutant DMPK mRNA in cytoplasm and in nuclei of DM1 myoblasts (Dr. Partha Sarkar, personal communication). Additional studies are required to examine nucleus/cytoplasmic distribution of the mutant DMPK mRNA by using different methodological approaches. This issue is very important for the development of therapeutic approaches to treat DM1.

### The Processing and Fate of the Mutant Intron 1 of ZNF9 in DM2 Cells

In DM2, expanded RNA CCUG repeats are located within intron 1 of *ZNF9* gene [8]. There are two main spliced isoforms of ZNF9 [49]. It was expected that the presence of CCUG expansion may interfere with splicing of ZNF9 pre-mRNA. However, two studies failed to detect splicing alterations for ZNF9 in DM2 [50,51]. It has been shown that, in DM2 myoblasts, the mutant CCUG RNA is aggregating in nuclei similar to the mutant DMPK mRNA in DM1 cells; however, these aggregates do not appear to contain sequences surrounding CCUG expansion [50]. These findings suggest that the pure CCUG repeats are accumulating in large aggregates after excision of mutant intron 1 of ZNF9. If this is the case, then the accumulation of CCUG repeats in large aggregates might lead to the reduction of decay of the mutant intron 1 of ZNF9 in DM2 cells.

Introns are degraded in nuclei by exosome immediately after their excision and linearization. The aggregation of CCUG repeats in DM2 nuclei suggests that there is a block or delay of the degradation of the mutant intron 1. However, investigations of the co-localization of CCUG foci and exosome have shown that CCUG repeats are not associated with exosome [46]. It is also possible that degradation of the mutant CCUG RNA repeats is reduced during excision of the mutant intron 1 at the stage of de-branching or linearization of intron. Our recent data with ectopic expression of RNA CCUG repeats in C2C12 myoblasts showed that short RNA CCUG repeats (CCUG_36_) also aggregate in nuclei and in cytoplasm [52]. These data suggest that the large number of copies of short RNAs CCUG repeats cannot be normally processed resulting in aggregation of CCUG_36_ RNA. The detection of foci containing 36 CCUG repeats in transfected C2C12 myoblasts might be also associated with a possible increase of their stability. It is likely that some RNA CCUG repeats in DM2 myoblasts escape nucleus and migrate to cytoplasm [52]. How CCUG repeats might reach the cytoplasm? One possibility is that nuclear CCUG repeats could migrate to cytoplasm during mitosis when nuclear membrane becomes fragile. In addition, there are some examples of decay of introns which occurs in cytoplasm [53]. It is important to note that cytoplasmic CCUG RNA repeats are found not only in proliferating DM2 myoblasts, but also in mature tissues such as liver of CCUG transgenic mice [52]. These data emphasize that the degradation of the mutant intron 1 of ZNF9 should be carefully examined to identify mechanism regulating its degradation.

### Toxicity of the Aggregated Forms of CUG and CCUG RNAs is Associated with Sequestration of Splicing Regulator, MBNL1

The aggregated forms of mutant DMPK mRNA are mainly detected in the nuclei of DM1 cells [26,31,32,37,38]. The nuclear CUGn aggregates sequester MBNL1 (Muscleblind) protein [31,54], causing local reduction of MBNL1. Analysis of MBNL1-knock out mice showed that homozygous animals developed main symptoms of DM1: myotonia, muscular dystrophy and cataracts [55]. Moreover, adenoviral delivery of MBNL1 into CUG-transgenic mice partially corrects myotonia [56]. These data show that sequestration of MBNL1 by nuclear CUG aggregates plays an important role in DM1 pathology. Since MBNL1 is also sequestered by nuclear aggregates containing CCUG repeats in DM2 cells, it might play similar role in DM2 pathogenesis. Because CCUG expansions are longer than CUG expansions, they might sequester large amounts of MBNL1. It remains to examine the relationship between the reduction of MBNL1 in DM2 and relatively mild phenotype of DM2.

In the course of studies of molecular pathogeneses of DM1 and DM2, it became clear that the mechanisms of these diseases are much more complex and are not limited to the alterations in splicing. Several recent reports suggest that the nuclear aggregates of the mutant DMPK mRNA are not sufficient to cause DM1 phenotype. Although the induction of the mutant 3’ UTR of DMPK in mice leads to accumulation of nuclear aggregates and to sequestration of MBNL1; these mice do not show overt DM1 phenotype [34]. In contrast, expression of a high number of copies of the DMPK 3’ UTR containing 5 CUG repeats leads to development of DM1-like phenotype (myotonia, cardiac conduction defects and muscular dystrophy) in the absence of nuclear CUG foci and in the absence of MBNL1 sequestration [34]. What is the mechanism by which multiple copies of the normal 3’ UTR of DMPK cause DM1 phenotype? Molecular analysis of the “inducible” transgenic mice with overt DM1 phenotype showed that these mice have increased levels of CUGBP1 (CUG-binding protein 1) [34]. Based on these results, it is reasonable to suggest that DM1 phenotype could be caused equally well by the reduction of MBNL1 and by the increase of CUGBP1. This suggestion is supported by several other *in vivo* models of DM1. In DM1 *Drosophila* model (generated in the Dr. Botas’ lab), interrupted CUG repeats (CUG_20_CUCGA_24_) cause muscle wasting and eye degeneration; and this phenotype is rescued by overexpression of MBNL1 [57]. The increase of CUGBP1 in muscle of *Drosophila* caused phenotype which is similar to that caused by overexpression of CUG repeats [57]. Importantly, flies overexpressing MBNL1 in the absence of intrerrupted CUG_480_ also show muscle phenotype, suggesting that MBNL1 levels have to be tightly regulated for normal muscle function. It is also interesting that flies with increased CUGBP1 crossed with flies expressing interrupted CUG_480 _RNA showed worsening eye degeneration suggesting that the increase of CUGBP1 levels is toxic for normal cells. The co-expression of CUGBP1 with interrupted CUG_480_ increases muscle wasting compared to flies expressing only CUG_480_ RNA [57].

**Fig. (4):**
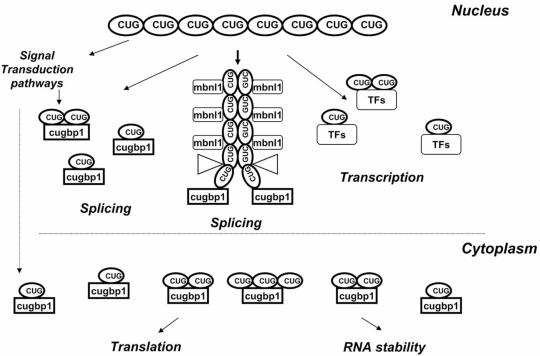
**A hypothetical model outlining the role of CUG expansion in the dys-regulation of gene expression at several levels.** *In the nucleus of DM1 patients*, double-stranded CUG repeat RNA binds to MBNL1. CUGBP1 binds to the opened ends of the CUG-helix. Triangles show hypothetical positions of other misregulated RNA-binding proteins, which interact with CUG repeats, such as hnRNP H. Un-aggregated CUG repeats bind to CUGBP1 and TFs affecting splicing of the CUGBP1 targets and reducing transcription. *In cytoplasm of DM1 cells*, the un-aggregated CUG repeats stabilize CUGBP1 increasing levels of CUGBP1 protein. The elevated CUGBP1 alters translation and stability of mRNAs. CUG repeats also change signal transduction pathways by unknown mechanisms affecting CUGBP1 activity and stability.

It is important to note that the increase of CUGBP1 causes degeneration in DM1 *Drosophila* model without binding to the aggregated form (foci) of mutant CUG_480_ RNA. This observation suggests that the elevation of CUGBP1 and reduction of MBNL1 might cause DM1 phenotype through independent mechanisms. One of these possible mechanisms has been proposed by Dr. Junghans [41]. It has been suggested that CUGBP1 and MBNL1 cause DM1 pathology through the binding to two different forms of mutant CUG RNA: “insoluble” or aggregated CUG repeats (MBNL1) and “soluble” or un-aggregated CUG RNA (CUGBP1) (Fig. **4**). According to this model, the mutant RNA with long CUG repeats exists in the double-stranded form, stability of which depends on the relative levels of free CUGBP1 and MBNL1. MBNL1 binds to the aggregated form of CUGn RNA, organized in the double-stranded helix; whereas CUGBP1 binds to the melted regions of the CUG helix [41]. In this case, saturation of the ds-CUG helix with MBNL1 reduces the levels of free MBNL1 facilitating melting of the ds-CUG helix and promoting CUGBP1 binding to the single stranded CUG repeat regions. This model is supported by electron microscopy data showing that CUGBP1 protein interacts with the base of the ds-CUG helix [58]. However, this model is not consistent with data showing that the elevation of CUGBP1 increases the number of CUG aggregates in transgenic flies suggesting that CUGBP1 does not melt, but rather stabilizes CUG aggregates [57]. Thus, the understanding of the relationships between CUGBP1 and MBNL1 in patients with DM1 requires more experimental work. More studies are also needed to determine the contribution of the reduction of MBNL1 and elevation of CUGBP1 in DM1 phenotype.

While CUG repeats in the *Drosophila* model, reported in [57] were toxic, another DM1 model has shown that the expression of CUG repeats is not toxic despite accumulation of CUG repeats in aggregated form and sequestration of muscleblind [59]. Several strains of transgenic flies expressing 16, 240 and 480 CUG repeats were generated by Dr. Ait-Ahmed’s group [60]. Surprisingly, only strain with 240 CUG repeats caused overt phenotype assessed by lethality and eye degeneration. Transgenic flies expressing 480 CUG RNA repeats were asymptomatic despite accumulation of nuclear CUG foci and sequestration of muscleblind [60]. This observation suggests that, in addition to the aggregation of CUG repeats and sequestration of muscleblind, other factors are involved in CUG RNA toxicity. Since the authors found the same levels of expression of RNA CUG repeats in both lines, the lack of phenotype in the 480 CUG repeats line could not be explained by the differences in the levels of CUG-containing transcripts. It is interesting that in the *Drosophila *line, showing degeneration, insertion occurred in the gene encoding zinc finger protein [60]. Thus, sequences surrounding CUG repeat expansion seem to be important for the toxicity of CUG repeats.

**Fig. (5):**
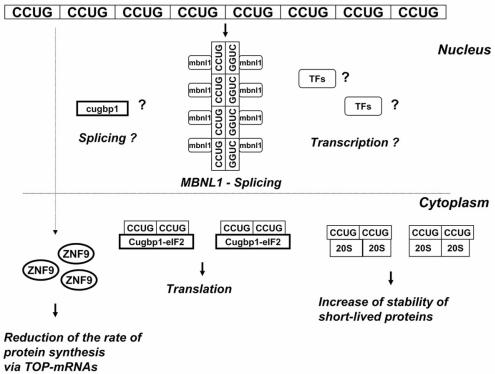
**Hypothetic model for the role of CCUG repeats in DM2 pathology**. Like in patients with DM1, CCUG repeats form nuclear aggregates presumably containing double-stranded CCUG repeats. This aggregated CCUG RNA sequesters MBNL1 changing splicing of mRNAs regulated by MBNL1. It is unknown if CUGBP1 contributes to the alterations in splicing of mRNAs in DM2 cells. *In cytoplasm*, the un-aggregated CCUG repeats bind to two multi-protein complexes containing CUGBP1-eIF2 and the 20S proteasome affecting translation and stability of proteins. CCUG repeats also reduce cytoplasmic levels of ZNF9 by unknown mechanism. Since ZNF9 regulates several TOP-containing mRNAs, encoding proteins of translational apparatus, the reduction of cytoplasmic ZNF9 causes the reduction of the rate of global protein synthesis.

The major toxicity of the mutant CUG and CCUG nuclear aggregates is associated with alterations of splicing of mRNAs regulated by MBNL1 (Fig. **4**, **5**) [31]. Analysis of splicing of some mRNAs in the DM1 *Drosophila *models generated by Dr. Artero’s group showed that there is a more complicated relationship between global splicing abnormalities and the sequestration of muscleblind. While transgenic flies with 480 CUG repeats showed stronger phenotype than flies expressing 60 CUG repeats, splicing abnormalities of some mRNAs were stronger in the line with lower number of CUG repeats [61]. Although the authors concluded that nuclear CUG foci may be not toxic (at least in *Drosophila)*, it is possible that short CUG repeats (CUG_60_) cause greater abnormalities in splicing of some mRNAs due to higher levels of expression. This suggestion is based on the overt DM1 phenotype in “tet-inducible” mouse model expressing high number of copies of the 3’ UTR of normal DMPK with 5 CUG repeats [34]. We have recently found that the expression of short CCUG repeats (CCUG_36_) in normal myoblasts causes changes in RNA processing identical to those observed in DM2 cells. These data support the suggestion that the high number of copies of short CCUG repeats have the same toxicity as low number of copies of long repeats [52]. In addition to aggregated CUG repeats in nuclei, the mutant CUG foci are detected in cytoplasm [47]. Whereas cytoplasmic CUG foci do not have toxic effect on splicing, they likely affect cytoplasmic processes in DM1 cells such as translation and RNA stability.

### Toxicity of Soluble CUG and CCUG Repeats is Mediated Through the Elevation of CUGBP1

One of the molecular hallmarks of DM1 is the elevation of CUGBP1 protein and its RNA-binding activity [34,39,62,63]. In patients with DM1, CUGBP1 protein is increased without significant changes of CUGBP1 transcripts levels [39]. The role of CUG repeats in the elevation of CUGBP1 in DM1 has been shown by examination of DM1 cellular and mouse models in which expression of CUG repeats led to the increase of CUGBP1 [34,39,63-65]. However, one DM1 mouse model did not show elevation of CUGBP1 [32]. It would be interesting to determine if CUG repeats elevate CUGBP1 RNA-binding activity in these transgenic mice.

The elevation of CUGBP1 has been also reported for DM2 patients [52]; however, there are contradictory results for expression of CUGBP1 in DM2. Two reports addressing total cellular levels of CUGBP1 in DM2 cells did not find differences in CUGBP1 levels [66,67]. However, analysis of cytopasmic extracts from DM2 cultured myoblasts and from muscle biopsies of DM2 patients showed the elevation of CUGBP1 [52]. Expression of pure RNA CCUG repeats in normal cells also increased levels of CUGBP1 [52]. It has been shown that the non-coding CCUG RNA in the DM2 mouse model generated by Dr. Krahe elevates CUGBP1 levels [52].

The mechanisms by which CUG and CCUG repeats elevate CUGBP1 need additional investigations. Since CUGBP1 has been not found in the nuclear CUG and CCUG aggregates, nuclear CUG and CCUG foci do not appear to affect CUGBP1 levels. On the contrary, identification of CUGBP1-RNA complexes from DM1 and DM2 cells by biochemical methods shows that CUGBP1 forms stable complexes with CUG and CCUG RNAs in DM1 and in DM2 correspondingly and these complexes are not detected in normal cells [39,52]. The formation of these complexes depends on CUG/CCUG RNAs because ectopic expression of pure CUG or CCUG RNAs causes interaction of CUGBP1 with un-aggregated CUG and CCUG repeats [39,52]. In agreement with these observations, the analysis of CUGBP1 in tissues from DM2 transgenic mice showed that CUGBP1 is associated with cytoplasmic CCUG RNA [52]. Immunoprecipitation of CUGBP1 from normal and DM1 cultured cells also showed strong interaction of CUGBP1 with the mutant DMPK mRNA in DM1 cells but not in normal cells [40]. These data suggest that CUGBP1 interacts *in vivo* with CUG and CCUG RNAs located outside of aggregated CUG and CCUG repeats (Fig. **4**, **5**). Since CUGBP1 mRNA levels are not increased in DM1 cells, it was suggested that CUG repeats may increase CUGBP1 stability. In fact, examination of CUGBP1 half-life in the presence of CUG repeats showed that CUG repeats stabilize CUGBP1 [39]. Recent data revealed that phosphorylation of CUGBP1 by PKC kinase also plays a significant role in the stabilization of CUGBP1 in DM1 cells [63]. Thus, stabilization of CUGBP1 in DM1 is achieved by a complex mechanism involving specific phosphorylation of CUGBP1, its interactions with un-aggregated CUG repeats, and possibly some other factors.

Growing number of new reports suggest that additional pathways are involved in the regulation of activity and levels of CUGBP1 in DM1 and DM2 cells. Analysis of CUGBP1 stability in DM2 myoblasts and in normal cells expressing CCUG repeats showed that the stability of CUGBP1 is also increased in the presence of CCUG repeats [52]. CUGBP1 phosphorylated isoforms are increased in cytoplasm of DM2 myoblasts with elevated levels of CUGBP1; thus phosphorylation may also play a role in the stabilization of CUGBP1 in DM2 cells [52]. In addition, it has been shown that the 20S proteasome is inhibited by CCUG repeats in DM2 myoblasts increasing stability of proteins [52]. Thus, the increase of CUGBP1 stability in DM2 patients may be also due to reduced activity of the 20S proteasome.

### Toxic Effect of CUGBP1 Elevation on the Global Splicing

Why the increase of CUGBP1 is toxic for cell functions? Detailed analysis of CUGBP1 in normal cells showed that this protein has many functions and plays an important role in several biological processes. CUGBP1 is expressed in both nucleus and cytoplasm. Like MBNL1, CUGBP1 has splicing activity [62-65,68-70]. CUGBP1 has also several important functions in cytoplasm, including regulation of protein translation and RNA stability [39,71-84]. Given these multiple functions, it is expected that the increase of CUGBP1 in DM1 and in some patients with DM2 might change splicing, translation and stability of mRNAs, targets of CUGBP1. It is interesting that CUGBP1 and MBNL1 regulate splicing of the same mRNAs (chloride channel 1, insulin receptor, troponin T) by binding to different sites [24,85]. This suggests that the increase of CUGBP1 or reduction of MBNL1 cause similar alterations of splicing in DM1 cells. However, transgenic mice overexpressing CUGBP1 developed splicing abnormalities suggesting that misregulation of splicing of CUGBP1 targets *in vivo* is independent of MBNL1 [68]. It is important to note that, in MBNL1 knock out mice, alteration of splicing also occurs without CUGBP1 increase [55]. It is likely that MBNL1 and CUGBP1 regulate splicing in a tissue specific manner contributing to different symptoms in patients with DM1 and DM2.

### Toxic Effect of CUGBP1 Elevation on Translation in DM1 and DM2 Cells

CUGBP1 regulates cap-dependent and cap-independent translation [71-78]. CUGBP1-dependent activation of translation is mediated by binding of CUGBP1 to mRNAs and to translation initiation eIF2 complex and following delivery of the mRNAs to polysomes [75]. Interactions of CUGBP1 with eIF2 require site-specific phosphorylation of CUGBP1 at Ser302 by cyclin D3-cdk4 kinase [75,76,78]. It has been shown that the interaction of CUGBP1 with eIF2 is increased during normal muscle differentiation [78]. It is important to note that, in DM1 myotubes, this interaction is reduced due to low levels of cyclin D3 in cytoplasm of DM1 cells [78]. As the result, translation of some mRNAs is reduced during differentiation of DM1 muscle [72,73,78]. Ectopic expression of cyclin D3 promoted the formation of the CUGBP1-eIF2 complex in DM1 myotubes and improved fusion of DM1 myoblasts [78]. These data show that CUGBP1 plays a critical role in the regulation of muscle differentiation. Consistent with this suggestion, modulation of CUGBP1 levels in animal models dys-regulates normal muscle development and differentiation. CUGBP1 transgenic mice so far as are the only one mouse model reproducing symptoms of CDM [73]. CUGBP1 transgenic mice with the levels of CUGBP1 matching those in CDM patients (approximately 5-fold) showed a delay in development and died in utero or shortly after birth [73]. Some of the underdeveloped pups have survived the postnatal stress only after additional supportive measures such as keeping underdeveloped pups separately with foster mothers up to 2 months of age until they are capable to eat dry food.

**Fig. (6):**
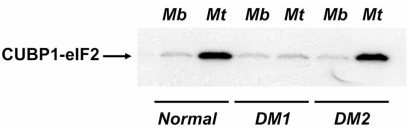
**The translational CUGBP1–eIF2 complex is increased in control and DM2 myotubes, but not in DM1 myotubes**. Cytoplasmic extracts from normal, DM1 and DM2 myoblasts (Mb) and myotubes (Mt) were incubated with the C/EBPβ RNA probe and separated by native gel electrophoresis (EMSA). The upper fragment of the gel containing the CUGBP1-eIF2 complexes is shown. The presence of CUGBP1-eIF2 complex in DM2 myotubes suggests that despite the increase of CUGBP1 in both DM1 and in DM2, CUGBP1 translational activity might be normal in DM2 myotubes.

In contrast to DM1 myotubes, CUGBP1-eIF2 complexes are increased in DM2 differentiating myotubes similar to normal myotubes (Timchenko, L.; unpublished; Fig. **6**). These data suggest that a lack of CDM in patients with DM2 may be, at least in part, due to normal phosphorylation of CUGBP1 at Ser302.

### Regulation of CUGBP1 RNA-Binding Activity by Phosphorylation

CUGBP1 has a dual effect on translation of mRNAs which depends on the efficiency of the binding of CUGBP1 to its targets. It has been shown that specificity of interaction of CUGBP1 with mRNAs is regulated by phosphorylation [75,76,78]. Two specific sites of phosphorylation [targeted by Akt (Ser28) and cyclinD3/cdk (Ser302)] were identified within CUGBP1 molecule [78]. Examination of CUGBP1 phosphorylation status in the DMPK knock out mice suggested that CUGBP1 is a possible substrate for DMPK kinase [86]. It has been also shown that PKC phopshorylation regulates CUGBP1 [63]. Although there are several predicted sites for the phosphorylation of CUGBP1 by PKC, additional studies are needed to identify phosphorylation sites for PKC and DMPK kinases within the CUGBP1 molecule.

**Fig. (7):**
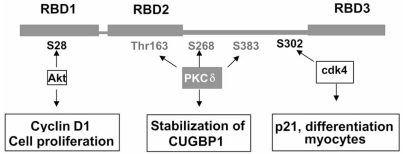
**The role of signal-transduction pathways in dysregulation of activity of CUGBP1 in patients with DM1.** CUGBP1 structure with three RNA-binding domains (RBD) is shown. Two sites (Ser28 and Ser302) phosphorylated by Akt and cyclin D/cdk4 correspondingly are shown. Amino acid residues predicted to be phosphorylated by PKCδ are also shown. Phosphorylation of CUGBP1 at Ser28 increases binding of CUGBP1 to cyclin D1 mRNA, promoting proliferation in myoblasts. Phosphorylation of CUGBP1 by PKCδ contributes to the stabilization of CUGBP1 in DM1. Site-specific phosphorylation of CUGBP1 by cdk4 increases interactions of the CUGBP1 with p21 mRNA leading to the elevation of p21 and promotion of skeletal muscle differentiation. In DM1 myogenesis, the reduction of cyclin D3 inhibits CUGBP1 binding to p21 mRNA and reduces p21 levels leading to a delay of DM1 differentiation.

It has been shown that the site-specific phosphorylation of CUGBP1 by Akt and cyclinD3/cdk4 kinase regulates CUGBP1 function during normal myogenesis (Fig. **7**). In proliferating myoblasts CUGBP1 is phosphorylated by Akt and the ph-S28-CUGBP1 has increased binding activity toward mRNA encoding cyclin D1 [78]. As noted above, in normal myotubes, CUGBP1 interacts with cyclin D3/cdk4 complex which phosphorylates CUGBP1 at Ser302 [68]. Ph-Ser302-CUGBP1 strongly binds to mRNA encoding a cdk inhibitor, p21, while binding of CUGBP1 to cyclin D1 mRNA is weaker. During DM1 myogenesis, phosphorylation of CUGBP1 and CUGBP1 interactions with its RNA targets are altered. In DM1 myoblasts, CUGBP1 is hyperphosphorylated by Akt; whereas in DM1 myotubes CUGBP1 phosphorylation by cyclinD3/cdk4 is reduced due to low levels of cytoplasmic cyclin D3 [68]. These changes of CUGBP1 phosphorylation in DM1 myogenesis lead to the increase of cyclin D1 in DM1 myoblasts and to the reduction p21 in DM1 myotubes [68] (Fig. **7**). Cyclin D1 is an important regulator of cell proliferation, while p21 a key regulator of the transition of dividing myoblasts to differentiation. Thus, changes of Akt-CUGBP1-cyclin D1 and cyclinD3/cdk4-CUGBP1-p21 pathways in DM1 disease might affect the efficiency of myogenesis causing a delay of differentiation. In addition, the phosphorylation of CUGBP1 on the putative PKC sites might stabilize CUGBP1 in DM1 cells leading to the enhancement of CUGBP1 functions. In summary, these data show that biological functions of CUGBP1 are altered in DM1 patients not only by the elevation of the protein, but also by phosphorylation-specific changes in RNA-binding activity of CUGBP1.

Elevation of CUGBP1 in DM2 muscle cells and tissues suggests that CUGBP1-dependent pathways might be also altered in DM2 cells similar to alterations observed in DM1. However, the DM2 phenotype is milder than DM1. Comprehensive analysis of CUGBP1 in DM2 cells and in DM2 models revealed several essential differences in CUGBP1 function in DM2 compared to DM1. CUGBP1 binds to CUG repeats within the DM1 protein extracts mainly as a single protein; however, in DM2 extracts, CUGBP1 binds to CCUG repeats as a component of the high molecular weight CUGBP1-eIF2 complex [52]. As noted above, CUGBP1-eIF2 complexes are increased in DM2 differentiating myotubes similar to normal myotubes (Fig. **6**); suggesting that in DM2 myotubes, phosphorylation of CUGBP1 at Ser302 is normal. If this is the case, then the RNA-binding activity of CUGBP1 toward its RNA targets might be different in DM1 and in DM2.

### Effects of Elevation of CUGBP1 in DM1 on Stability of mRNAs 

Growing number of evidence indicates that biological functions of CUGBP1 are much broader than it has been initially suggested. Recent studies demonstrated that CUGBP1 is involved in the regulation of stability of short-lived mRNAs (cytokines and oncogenes) through the binding to the GRE (GU-rich) elements in their 3’ UTRs [83,84]. A large number of CUGBP1 mRNAs targets, stability of which may be controlled by CUGBP1, has been identified [80,83,87]. Several identified mRNAs control multiple biological processes in cells. Among those is mRNA encoding cytokine TNF (Tumor Necrosis Factor) [80,87]. It has been shown that CUGBP1 binds to the 3’ UTR of TNF mRNA and destabilizes it through the direct interaction with poly A ribonuclease (PARN) removing the poly(A) tail [80]. Consistent with these findings, expression of the mutant DMPK in C2C12 myoblasts increases stability of TNF mRNA [87]. As the result, TNF is elevated in transfected cells. TNF alpha levels are also increased in sera from DM1 patients [88]. Increase of TNF alpha in DM1 could be due to inflammation associated with dystrophic muscle; but it also could be due to increased levels of TNF through the dysregulation of CUGBP1. Such dysregulation of RNA stability by CUGBP1 may contribute to DM1 phenotype at different levels, including muscle wasting and insulin resistance.

CUGBP1 is homologous to the *Xenopus* EDEN-BP protein which regulates RNA deadenylation through EDEN element during development [79,81]. Given these additional activities of CUGBP1, it is not surprising that lower or high levels of expression of CUGBP1 have a toxic effect on normal development [73,89]. Identification of all CUGBP1 mRNA targets involved in splicing, translation and RNA stability is the next important step to determine the toxicity of CUG/CCUG repeats associated with the elevation of CUGBP1.

### Toxicity of CUG Repeats Might be Associated with the Reduction of Transcription Factors and with Alteration of Chromatin Remodeling Proteins

In addition to alterations of RNA-binding proteins, the mutant CUG repeats affect transcription factors (TFs) by leaching Specificity protein 1 (Sp1) and Retinoic Acid Receptor (RAR) out of active chromatin [40]. These TFs were identified in the RNPs containing mutant but not normal DMPK mRNA. As the result, these TFs are reduced in DM1 cells [40]. Like CUGBP1, TFs do not bind to the aggregated form of mutant CUG RNA, suggesting that they are affected by un-aggregated CUG repeats (Fig. **4**). It remains to investigate if the mutant CCUG repeats affect certain TFs in patients with DM2. It has been suggested that global transcription in DM1 may be affected by mutant CUG repeats indirectly through alterations of splicing [90]. However, other mechanisms misregulating transcription in DM1 and DM2 are possible. In addition to the reduction of TFs due to binding to soluble CUG repeats, transcription may be changed at the levels of chromatin remodeling. Our recent data showed that CUGBP1 regulates translation of histone deacethylase 1 (HDAC1) [91]. This suggests that elevation of CUGBP1 in DM1 and DM2 patients might increase HDAC1 levels which in turn might alter transcription of many genes. Further studies are required to test this possibility.

## IDENTIFICATION OF MRNAS REGULATED BY CUGBP1 AND MBNL1 IS REQUIRED FOR THE DEVELOPMENT OF THERAPY FOR DM

Development of approaches reducing toxicity of CUG and CCUG repeats requires a better understanding of the primary targets of CUG/CCUG repeats. Data discussed above suggest that CUG and CCUG repeats affect MBNL1 and CUGBP1 independently through aggregated and un-aggregated CUG and CCUG repeats. Early elevation of CUGBP1 in transgenic mice expressing CUG repeats shows that the increase of CUGBP1 is not a consequence of different abnormalities in DM1 but rather a direct result of expression of the mutant CUG repeats [64]. Evaluation of contribution of CUGBP1 and MBNL1 in DM1 and DM2 pathologies would require identification of mRNAs which are regulated by these proteins *in vivo*. Many attempts have been made to determine MBNL1 and CUGBP1 binding sites within mRNAs: natural targets of these proteins. So far, the usage of numerous methods *in vitro* has produced contradictory observations. It has been initially suggested that MBNL1 binds exclusively to the double-stranded structures formed by long CUG repeats [31]. However, recent reports found that MBNL1 binds not to only long repeats, but also to short repeats comprising of CUG as well as CAG repeats [92,93]. Moreover, MBNL1 is sequestered into nuclear foci formed by both CAG and CUG repeats [44]. In addition, the initial concept that only long CUG repeats form double-stranded RNA structures has been corrected by recent data showing that six CUG repeats (CUG_6_) are sufficient to form double-stranded helix [94].

CUGBP1 has been identified as the protein which binds to RNA oligonucleotide containing eight CUG repeats (CUG_8_) [27,28]. CUGBP1 has three RNA-binding domains (RBDs). Further studies showed that RBD1+2 interact with CUG repeats; while RBD3 of CUGBP1 binds to GCN-rich regions in the 5’ UTRs of mRNA targets [ref. 71, and Timchenko L., unpublished]. It has been later shown that CUGBP1 also binds to U(A/G) repeat and to GRE elements [79,81,83]. As has been discussed above, CUGBP1 does not bind to aggregated CUG and CCUG repeats.

It seems that the best way to determine biologically relevant binding sites for these proteins is to identify mRNAs which are associated with CUGBP1 and MBNL1 *in vivo*, particularly in normal and in DM cells. A comprehensive analysis of CUGBP1-RNPs from the CUGBP1 transgenic and MBNL1-RNPs from the wild type and MBNL1 knock out mice would be one of the approaches for identification of mRNAs which are targets of CUGBP1 and MBNL1 *in vivo*. In the case of CUGBP1, it is clearly shown that this protein has multiple targets with a variety of binding sites [27,39,69-76]. Analysis of the targets of CUGBP1 and MBNL1 will help to determine the role of each of these proteins in DM1 and DM2 pathologies.

## PROTEIN TURNOVER IN DM2

Although initial studies suggested that DM2 pathology is mainly mediated by changing in alternative splicing, further studies showed much more complex mechanisms for DM2. Examination of cytoplasmic RNA-protein complexes binding to CCUG repeats revealed that un-aggregated CCUG repeats sequester the 20S proteasome [52]. In agreement with the sequestration of the 20S proteasome, the stability of short-lived proteins is increased in DM2 cells [52]. These data suggested that the mutant CCUG repeats outside of the CCUG foci may alter protein metabolism in DM2 patients (Fig. **5**). In addition, DM2 cells contain abundant translational CUGBP1-eIF2 complex which changes translation of certain proteins in DM2 cells. Interestingly, the 20S proteasome complex in DM2 cells is associated with ER chaperone BiP, which is a master regulator of Unfolded Protein Response (UPR). Usually, the UPR signaling prevents protein aggregation by two pathways: (1) reduction of translation; and by (2) activation of splicing of a specific b-ZIP transcription factor, XBP1, which promotes transcription of genes regulating protein degradation. The presence of ER chaperones in the CCUG-binding multi-protein complexes and the accumulation of undegraded proteins in DM2 cytoplasm suggest that ER chaperones play a specific role in the attenuation of protein translation, RNA splicing and RNA expression in DM2. Thus, ER chaperones may have an additional toxic effect in DM2 cells.

Although majority of data pointed that CCUG expansion in the ZNF9 gene has a trans effect on gene expression, data *in vivo* show that ZNF9 deletion causes main symptoms of DM2 [95]. Since ZNF9 protein has been implicated in the regulation of cap-dependent and cap-independent translation through the binding to the 5’ UTRs of mRNAs, it has been suggested that ZNF9 might be a candidate to be involved in the mis-regulation of protein translation in DM2 [96-98]. Consistent with this hypothesis, a recent report has shown that ZNF9 interacts with the 5’ UTRs of TOP (terminal oligopyrimidine tract) mRNAs that encode proteins of translational apparatus: human ribosomal protein, RPS17, poly(A)-binding protein, PABP, and elongation factors, eEF1A and eEF2 [96]. The binding activity of ZNF9 toward the TOP-containing 5’ UTRs is significantly reduced in DM2 muscle. Decrease of proteins of translational apparatus in DM2 correlates with reduction of a rate of global protein synthesis in DM2 suggesting that CCUG toxicity is also associated with the inhibition of the rate of global protein translation in DM2 muscle cells.

## COMPARISON OF TOXICITY OF SHORT AND LONG CUG AND CCUG REPEATS

It has been shown that long CUG repeats in DM1 cells are cleaved by a dicer leading to accumulation of RNA containing short CUG repeats [99]. What are the biological consequences of accumulation of short CUG repeats? One possibility is that short CUG repeats may act as siRNAs regulating the levels of other genes [99]. Another possibility came from several recent observations which suggested that high number of copies of normal size of CUG or CCUG repeats are as toxic as low number of copies of long CUG and CCUG repeats. In DM1 mouse model generated by Dr. Korneluk’s lab, the expression of normal and mutant 3’ UTRs of DMPK caused a delay of muscle differentiation and muscle atrophy [100]. Elevation of normal 3’ UTR in the DM1 “inducible” transgenic mice caused myotonia, cardiac defects and muscular dystrophy [34]. Finally, ectopic expression of short CCUG repeats in normal muscle cells increases stability of proteins and increases translation of targets of CUGBP1 similar to DM2 cells with long expansions [52]. These new data suggest that incomplete degradation of expanded DMPK mRNA and mutant intron 1 of ZNF9 may produce large number of short CUG and CCUG repeats which are toxic products and which may increase the severity of the disease phenotype. Thus, searching for the therapeutic treatments of DM1 and DM2, it would be important to develop approaches for the complete elimination of both long and short CUG and CCUG repeats.

## CONCLUSIONS

1. The toxicity of CUG/CCUG repeats in DM is mediated by following mechanisms: a) reduction of MBNL1 in nuclei of DM1 and DM2; b) elevation of CUGBP1 in DM1 and DM2; c) alteration of splicing; d) increase of CUGBP1 translational targets; e) alteration of RNA stability; f) reduction of the rate of protein translation; g) reduction of TFs; h) increase of protein stability; i) increase of Akt and PKC kinases and k) the reduction of cyclin D3.
2. The length of CUG/CCUG expansions is critical; however, high number of copies of the short CUG and CCUG repeats might be also pathogenic.
3. Aggregation of CUG and CCUG repeats in nuclei might be toxic; however, additional studies are needed to examine the correlation of toxicity of the total amounts of CUG/CCUG RNA repeats with a number of CUG/CCUG nuclear aggregates.
4. Disruption of nuclear CUG foci with anti-sense to CUG RNA helps to correct MBNL1-dependent splicing in nuclei of DM patients [101,102]. The effect of anti-sense on cytoplasmic dysfunctions should be also tested.
5. Based on the current knowledge, the “ideal” approaches for DM therapy should include the efficient degradation of the mutant RNAs without disruption of the wild type DMPK and ZNF9 mRNAs. Such approaches would help to eliminate complex effects of CUG/CCUG repeats on molecular processes in DM tissues.

## Figures and Tables

**Table 1:** Genetic Etiology of DM1 and DM2

DM1		DM2
Chromosomal locus	19q 13.3	3q 21.3
Gene	*DMPK *	*ZNF9 *
Inheritance	autosomal dominant	autosomal dominant
Mechanism	CTG repeat expansion	CCTG repeat expansion
Normal repeat size	up to 37	up to 27
Pathologic repeat size	> 50 CTG	> 75 CCTG?
Expanded repeat range	50-4000	75-5000->11000 CCTG
Anticipation	yes	---
